# Proximity labeling of host factor ANXA3 in HCV infection reveals a novel LARP1 function in viral entry

**DOI:** 10.1016/j.jbc.2024.107286

**Published:** 2024-04-16

**Authors:** Hanna Bley, Christoph Krisp, Anja Schöbel, Julia Hehner, Laura Schneider, Miriam Becker, Cora Stegmann, Elisa Heidenfels, Van Nguyen-Dinh, Hartmut Schlüter, Gisa Gerold, Eva Herker

**Affiliations:** 1 Institute of Virology, Philipps-University Marburg, Marburg, Germany; 2 Section Mass Spectrometry and Proteomics, University Medical Center Hamburg-Eppendorf, Hamburg, Germany; 3 Institute for Biochemistry & Research Center for Emerging Infections and Zoonoses (RIZ), University of Veterinary Medicine Hanover, Hanover, Germany; 4 Department of Clinical Microbiology, Virology, Umeå University, Umeå, Sweden; 5 Wallenberg Centre for Molecular Medicine (WCMM), Umeå University, Umeå, Sweden

**Keywords:** Hepatitis C virus (HCV), proteomics, proximity labeling, lipid droplets, host-pathogen interaction, virus entry

## Abstract

Hepatitis C virus (HCV) infection is tightly connected to the lipid metabolism with lipid droplets (LDs) serving as assembly sites for progeny virions. A previous LD proteome analysis identified annexin A3 (ANXA3) as an important HCV host factor that is enriched at LDs in infected cells and required for HCV morphogenesis. To further characterize ANXA3 function in HCV, we performed proximity labeling using ANXA3-BioID2 as bait in HCV-infected cells. Two of the top proteins identified proximal to ANXA3 during HCV infection were the La-related protein 1 (LARP1) and the ADP ribosylation factor-like protein 8B (ARL8B), both of which have been previously described to act in HCV particle production. In follow-up experiments, ARL8B functioned as a pro-viral HCV host factor without localizing to LDs and thus likely independent of ANXA3. In contrast, LARP1 interacts with HCV core protein in an RNA-dependent manner and is translocated to LDs by core protein. Knockdown of LARP1 decreased HCV spreading without altering HCV RNA replication or viral titers. Unexpectedly, entry of HCV particles and E1/E2-pseudotyped lentiviral particles was reduced by LARP1 depletion, whereas particle production was not altered. Using a recombinant vesicular stomatitis virus (VSV)ΔG entry assay, we showed that LARP1 depletion also decreased entry of VSV with VSV, MERS, and CHIKV glycoproteins. Therefore, our data expand the role of LARP1 as an HCV host factor that is most prominently involved in the early steps of infection, likely contributing to endocytosis of viral particles through the pleiotropic effect LARP1 has on the cellular translatome.

Hepatitis C virus (HCV) infection is a major cause of liver cirrhosis and hepatocellular carcinoma. Globally, it is estimated that 58 million people are chronically infected and ∼0.3 million die annually due to HCV-induced liver cirrhosis and hepatocellular carcinoma (1). As treatment, pan-genotypic direct-acting antivirals are recommended that can cure more than 95% of HCV patients. However, most HCV infections are not noticed, antiviral treatment remains expensive in many countries, and there is no vaccine available. HCV is a bloodborne virus and belongs to the Flaviviridae family. It is an enveloped positive-sense ssRNA virus and is associated with lipoproteins and neutral lipids forming infectious lipoviroparticles (LVP) (2, 3, 4). The virus enters the cell by receptor binding and clathrin-mediated endocytosis, followed by a fusion step with low pH in the endosome (5, 6). The viral genome is released into the cytoplasm and translated into one single polyprotein, which is subsequently processed by cellular and viral proteases into the three structural proteins (the capsid protein core and the envelope glycoproteins E1 and E2) and the seven nonstructural proteins (p7, NS2, NS3, NS4A, NS4B, NS5A, and NS5B) (7). HCV RNA replication is localized at characteristic membrane vesicles, mainly double membrane vesicles, that originate from ER-membrane rearrangements (8). Replication vesicles containing the viral proteins NS3–NS5B are in close proximity to cytosolic lipid droplets (LDs) (9, 10). LDs act as viral assembly platforms for HCV and the recruitment of core and NS5A to LDs is essential for efficient particle production (11, 12, 13). Additionally, host proteins located at the LD surface–like perilipin (PLIN) 2 and 3 are crucial for virus production (14, 15, 16). Upon HCV infection, the LD proteome changes considerably. A previous quantitative LD proteome analysis revealed that annexin A3 (ANXA3) relocates to LDs during HCV infection and is critical for the incorporation of apolipoprotein (APO) E into LVPs *via* its interaction with E2 (17). Following up on these results, we performed ANXA3 proximity labeling analyses, using the biotin ligase BioID2 (18). BioID2 is based on a genetically modified promiscuous bacterial biotin ligase BirA that biotinylates proximate proteins within a 10 nm radius (19, 20). We first characterized the ANXA3-BioID2 construct and identified ANXA3-proximate proteins in hepatoma cells. Then, using quantitative proteomics, we compared HCV-infected ANXA3-BioID2–expressing cells with uninfected cells. The La-related protein (LARP) 1 and the ADP ribosylation factor-like protein (ARL) 8B were two of the top hits identified in close proximity of ANXA3 in HCV-infected cells.

A recent study reported that ARL8B is upregulated in HCV-infected cells and is involved in an HCV-induced autophagic block that is important for virion secretion (21). Overexpression of ARL8B causes a redistribution of lysosomes to the cell periphery, which blocks the autophagosome-lysosome fusion. Consequently, ARL8B knockdown reduces virion secretion by restoring normal autophagosome-lysosome fusion without affecting HCV RNA replication (21). Further, ARL8B plays a crucial role in mediating triglyceride remobilization from LDs to lysosomes by interaction with both organelles (22).

LARP1 has a conserved La domain and an RNA-binding region (23, 24). It is a major target downstream of the mechanistic (also known as mammalian) target of rapamycin complex (25, 26, 27). By binding to the 5′ motif of terminal oligopyrimidine (TOP) mRNAs, LARP1 stabilizes and regulates their translation (26, 28, 29, 30, 31, 32). LARP1 was identified as a biomarker in ovarian, lung, and hepatocellular cancer (33, 34). Previous proximity-based mapping of stress granules (SGs) and processing bodies revealed LARP1 in close proximity to the SG-associated protein polyadenylate-binding protein 1 (PABPC1) (35). LARP1 and PABPC1 interact within a ribonucleoprotein (RNP) complex (36, 37). Interestingly, it was reported that RNP complexes such as SGs and processing bodies are located proximal to LDs in HCV-infected cells (38, 39, 40, 41). In the context of HCV infection, LARP1 relocates to core-containing LDs, as part of an RNP complex (42) and associates with particles (43). However, the exact role of LARP1 in HCV infection has been contradictory: There is consensus that LARP1 localizes to core-coated LDs in HCV-infected cells and silencing LARP1 expression impairs HCV infection. However, mechanistically, LARP1 knockdown either increased or decreased the number of infectious particles released into the supernatant (42, 43). Therefore, we revisited the role of ARL8B and LARP1 and their interplay with ANXA3 at LDs during HCV infection.

## Results

### Establishment of an ANXA3-BioID2 proximity biotinylation system

**Figure 1:**
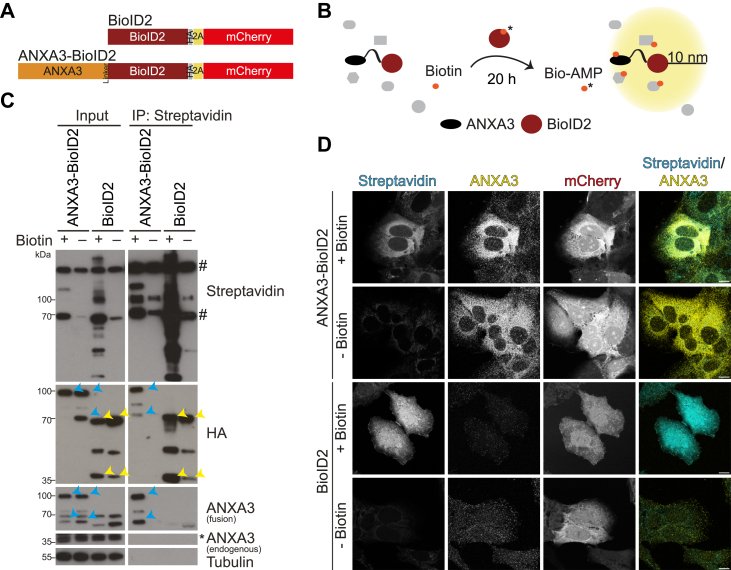
**Validation of ANXA3-BioID2 fusion construct.***A* and *B*, scheme of the BioID2^HA-2A-mCherry^ and ANXA3-BioID2^HA-2A-mCherry^ constructs (*A*) and biotinylation (*B*). *C*, BioID2^HA-2A-mCherry^- or ANXA3-BioID2^HA-2A-mCherry^-transduced Huh7.5 cells were labeled with biotin, lysed, and biotinylated proteins were purified with streptavidin-conjugated agarose beads and analyzed by immunoblotting. Biotinylated proteins were detected with streptavidin-HRP. Expression of ANXA3-BioID2 and BioID2 was analyzed using ANXA3 and/or HA-specific antibodies and tubulin served as a loading control. *Blue arrowheads* mark ANXA3-BioID2 and *yellow arrowheads* mark BioID2 proteins. *Asterisks* indicate unspecific bands and # indicate endogenously biotinylated proteins (representative blots of two independent experiments). *D*, immunofluorescence staining of biotinylated proteins using a streptavidin-conjugated fluorophore. ANXA3 was visualized using an ANXA3-specific antibody and mCherry served as a transduction control. Scale bar represents 10 μm (representative microscopy images of three independent experiments). ANXA3, annexin A3.

In previous studies, we have shown that the LD proteome is profoundly altered during HCV infection (17). ANXA3 was identified as a host factor for HCV that is recruited to LDs in HCV-infected cells and is required for HCV particle production by affecting the incorporation of APOE into LVPs (17). Apart from ANXA3, numerous cellular proteins relocalized to LDs in HCV-infected cells compared to naïve cells (17). To further investigate ANXA3-dependent HCV morphogenesis, we wanted to identify ANXA3 interactors or proximal proteins in HCV-infected cells. We genetically fused the bait protein ANXA3 to BioID2 followed by a 2A ribosomal skipping sequence and an mCherry fluorescent reporter (Fig. 1*A*). As a control, we used a BioID2 expression cassette (Fig. 1*A*). The fusion proteins were cloned into lentiviral vectors to enable stable expression. BioID2-based proximity labeling was performed by incubating cells with 10 μM biotin for ∼20 h (Fig. 1*B*). To validate the biotinylation system, we transduced Huh7.5 cells with lentiviral particles encoding ANXA3-BioID2 or BioID2, treated the cells with biotin and, after lysis, captured biotinylated proteins by pull down with streptavidin-conjugated agarose beads. Samples were then subjected to immunoblotting (Fig. 1*C*). Streptavidin-HRP detected two prominent bands in all samples, representing endogenously biotinylated mitochondrial proteins that are frequently observed in biotinylation-based proximity assays (44). Extensive biotinylation was detected in biotin-treated samples, with BioID2 biotinylating more proteins than ANXA3-BioID2. This reflects the more restricted localization of the ANXA3 fusion protein. Expression of ANXA3-BioID2 (Fig. 1*C*, blue arrowheads) and BioID2 (Fig. 1*C*, yellow arrowheads) was confirmed by ANXA3 and/or HA-specific antibodies. In both samples, bands with an additional molecular weight of ∼35 kDa were detected, indicating inefficient skipping of the 2A site; the respective bands at higher molecular weight likely represent mCherry-fused proteins. Overexpressed ANXA3-BioID2 was pulled down upon labeling *via* streptavidin, but not endogenous ANXA3. The BioID2 control was detected in the streptavidin pull-down of biotin-treated and to a lesser extent of the untreated samples.

Subcellular localization of the ANXA3 as well as biotinylation was additionally analyzed by immunofluorescence microscopy of ANXA3-BioID2- or BioID2-transduced cells that were either biotinylated or left untreated (Fig. 1*D*). mCherry, which served as a marker for transduction, displayed an unspecific localization with strong signals in the nucleus. Staining with a streptavidin-conjugated fluorophore indicated successful biotinylation of cytosolic proteins in biotin-treated ANXA3-BioID2–expressing cells and ubiquitous biotinylation in BioID2-expressing cells. Levels of biotinylated proteins were much higher in the biotinylated samples (Fig. 1*D*).

**Figure 2:**
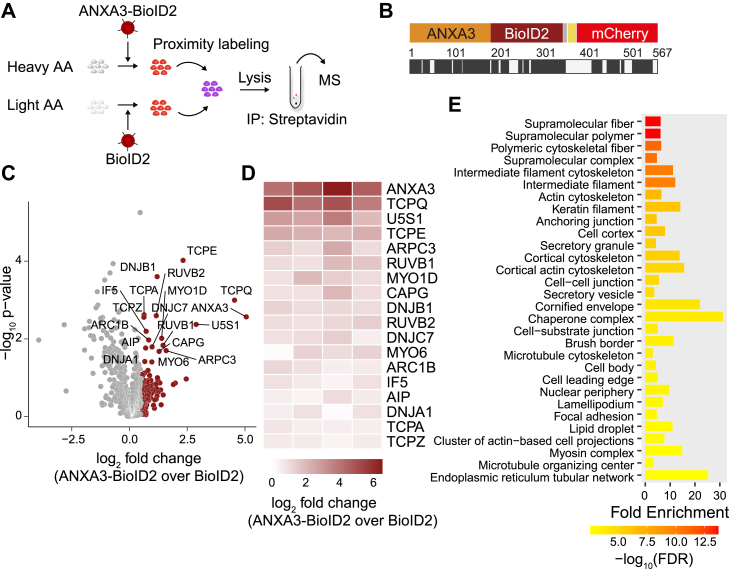
**Identification of ANXA3-proximal proteins using BioID2.***A*, scheme of the experiment. Huh7.5 cells cultured in media containing heavy or light amino acids were transduced with lentivirus expressing either ANXA3-BioID2 or BioID2. Cells were labeled with biotin, biotinylated proteins were pulled down using streptavidin-conjugated agarose beads, and subjected to MS. *B*, illustration of the peptide coverage of ANXA3-BioID2 fusion proteins using MS; *black* indicates peptide coverage. *C*, volcano plot of proteins identified in streptavidin pull downs of ANXA3-BioID2–expressing Huh7.5 cells over BioID2-expressing cells. Highlighted in *red* are enriched proteins (log_2_ fold change > 0.5) and proteins that are significantly enriched are labeled (unpaired two tailed *t* test with unequal variance (Welch’s *t* test) *p* ≤ 0.05). *D*, heatmap of significantly enriched proteins in ANXA3-BioID2–expressing cells compared to BioID2-expressing cells; columns represent independent experiments (log_2_ fold change > 0.5, unpaired two tailed *t* test with unequal variance (Welch’s *t* test) *p* ≤ 0.05, n = 4). *E*, GO analysis of ANXA3-proximate proteins. Bar graph of top 30 overrepresented cellular components terms of enriched proteins (log_2_ fold change > 0.5) with a *p*-value (FDR) cutoff of 0.05. Shown are the number of genes sorted by log_10_ (FDR). ANXA3, annexin A3; GO, gene ontology.

For quantitative analysis, we then performed stable isotope labeling by amino acids in cell culture (SILAC) followed by proximity biotinylation (Fig. 2*A*). Heavy or light amino acid–labeled Huh7.5 cells were transduced with either ANXA3-BioID2- or BioID2-carrying lentiviral particles and treated with biotin. Label-switch conditions were used in two out of four experiments. Lysates of heavy and light labeled cells were mixed, biotinylated proteins were pulled down by streptavidin beads, and samples were subjected to mass spectrometric proteomics. Multiple peptides spanning the ANXA3-BioID2 fusion protein were detected by mass spectrometry (Fig. 2*B*).

For relative quantification, signal intensity ratios of monoisotopic peaks of light over heavy peptides or heavy over light peptides for the label-switch were centered by dividing through the median. In total, 104 proteins were enriched (log_2_ fold change >0.5) in pull downs of ANXA3-BioID2- compared to BioID2-expressing cells (Fig. 2*C*, red color, Table S1). Next, significantly enriched hits were selected (*p* ≤ 0.05) (Fig. 2*D*). Gene ontology (GO) analysis of enriched proteins revealed that most proteins relate to components of the cytoskeleton (Fig. 2*E*). Specifically, proteins of the actin-related protein complex 2/3 (ARPC3 and ARC1B), profilin 1 (PROF1), which binds actin (45), as well as several T-complex protein 1 components (TCPQ, TCPE, TCPA, TCPZ, and TCPB) were enriched in close proximity to ANXA3 (Fig. 2*D*, Table S1). This chaperonin-containing T-complex (TRiC) was reported to play a role in actin and tubulin folding (46, 47). Notably, members of the annexin family are known regulators of actin dynamics (47, 48). It has been reported that annexins are recruited to actin-rich membrane subdomains and actin assembly points (49, 50). We also identified the Ras-related protein Rab21, an endosome-located protein, which is important for endocytic trafficking (51). In agreement with that, several annexins, including ANXA3, were found at early endosomes (52). Overall, our identified ANXA3-proximate proteome reflects previous reports on annexin-related functions.

### Identification of ANXA3-proximate proteins in HCV-infected cells

In order to study the proteome in HCV-infected cells *via* proximity labeling, we transduced Huh7.5 cells with lentivirus expressing BioID2^HA-2A-mCherry^ (Fig. 1*A*), infected with a Jc1^NS5AB-EGFP^ reporter construct (53) or left uninfected (Fig. S1*A*). Again, label-switch conditions were used in two out of four independent experiments. When >90% of the cells were HCV-positive (∼3 weeks p.i.), cells were labeled with biotin for 20 h, lysed, and biotinylated proteins were pulled down using streptavidin-conjugated agarose resins. Precipitated proteins were subjected to mass spectrometric proteomics. Next, signal intensity ratios of monoisotopic peaks of light over heavy peptides or heavy over light peptides for label-switch conditions were centered by dividing through the median. Transduction of the BioID2 ligase alone leads to ubiquitous biotinylation in the cell and serves as a background control of HCV-infected cells compared to uninfected cells. We identified several cytoplasmic proteins that were significantly altered upon infection (Fig. S1*B*). GO analysis showed that mostly proteins involved in the host cells translation machinery were identified (Fig. S1*C*).

**Figure 3:**
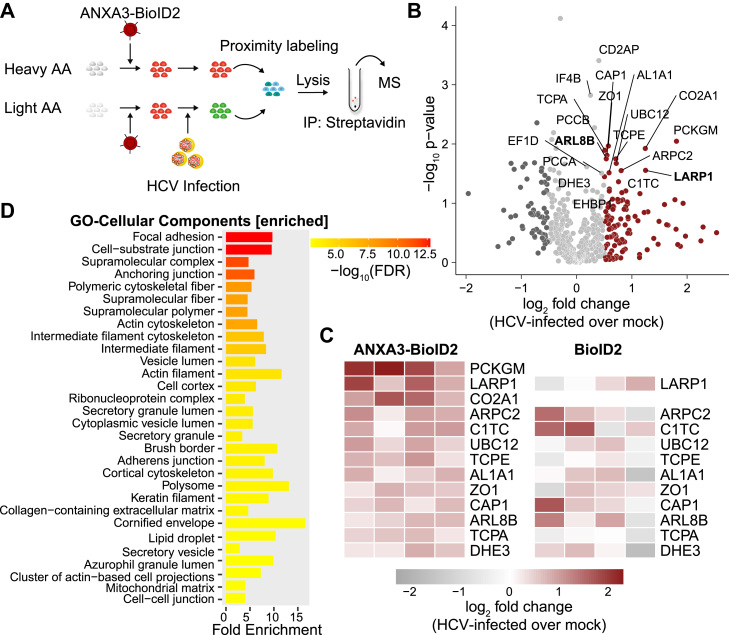
**Proteins identified by ANXA3-BioID2 proximity labeling in HCV-infected cells.***A*, scheme of the experiment. Huh7.5 cells cultured in media containing heavy or light amino acids were transduced with lentivirus expressing ANXA3-BioID2 and infected with a Jc1^NS5AB-EGFP^ reporter strain (MOI 0.01). Three weeks post infection, cells were incubated with biotin and biotinylated proteins were pulled down using streptavidin-conjugated agarose beads and subjected to MS. *B*, volcano plot of ANXA3-proximate proteins in HCV-infected over uninfected cells. Highlighted in *red* and *dark gray* are proteins enriched and depleted proximal to ANXA3 in HCV-infected cells compared to control (log_2_ fold change > 0.5 or < −0.5). Proteins that are significantly enriched are labeled (unpaired two tailed *t* test with unequal variance (Welch’s *t* test) *p* ≤ 0.05). *C*, heatmap of proteins significantly enriched proximal to ANXA3 in HCV-infected cells compared to control; columns represent independent experiments (log_2_ fold change > 0.5 or < −0.5, unpaired two tailed *t* test with unequal variance (Welch’s *t* test) *p* ≤ 0.05, n = 4). On the *right*, heatmap of the respective hits as found in proximity to only BioID2-expressing infected compared to uninfected cells; columns represent independent experiments (n = 4). *D*, GO analysis of ANXA3-proximate proteins enriched in HCV-infected cells. Bar graph of top 30 overrepresented cellular components terms of enriched or depleted proteins (log_2_ fold change > 0.5 or < −0.5) with a *p*-value (FDR) cutoff of 0.05. Shown are the number of genes sorted by log_10_ (FDR). ANXA3, annexin A3; GO, gene ontology; HCV, hepatitis C virus; MOI, multiplicity of infection.

To compare ANXA3 interactors in HCV-infected with uninfected cells, we performed SILAC experiments as described above with Huh7.5 cells that were transduced with lentivirus expressing ANXA3-BioID2^HA-2A-mCherry^ and infected with a Jc1^NS5AB-EGFP^ reporter construct (53) or mock-infected as described before (Fig. 3*A*). Of 543 proteins that were detected by multiple peptides in all four independent experiments, 13 were significantly (*p* ≤ 0.05) increased (log_2_ fold change > or <0.5) in their amount in HCV-infected cells compared to uninfected cells (Fig. 3, *B* and *C*, Table S1). Comparison of the fold change of significantly enriched hits in infected ANXA3-BioID2^HA-2A-mCherry^ cells to the mean values in infected BioID2^HA-2A-mCherry^ cells, we observed a lower fold change for all enriched proteins (Fig. 3*C*). This validates a specific enrichment of ANXA3-proximate proteins in HCV-infected cells, rather than an upregulation merely due to HCV infection. GO analysis of enriched proteins revealed overrepresented cellular compartments, especially cytoskeleton components, LDs, and RNP complexes (Fig. 3*D*). Two of the top ANXA3-proximal proteins enriched in HCV-infected cells were LARP1 and ARL8B. Both LARP1 and ARL8B were previously described as HCV host factors (21, 42, 43). Interestingly, ARL8B was also identified in our previous LD proteome analysis of HCV-infected cells (17). However, the exact roles during HCV infection remain unclear. ARL8B was shown to suppress lysosomal degradation of virions, thereby indirectly enhancing viral particle release (21). Recently, it was reported that ARL8B mediated LD turnover by binding to lysosomes and LDs (22). For LARP1, published results are contradictory, suggesting both a direct positive and negative function in HCV particle secretion (42, 43).

### LARP1 but not ARL8B localizes to LDs in HCV-infected cells

**Figure 4:**
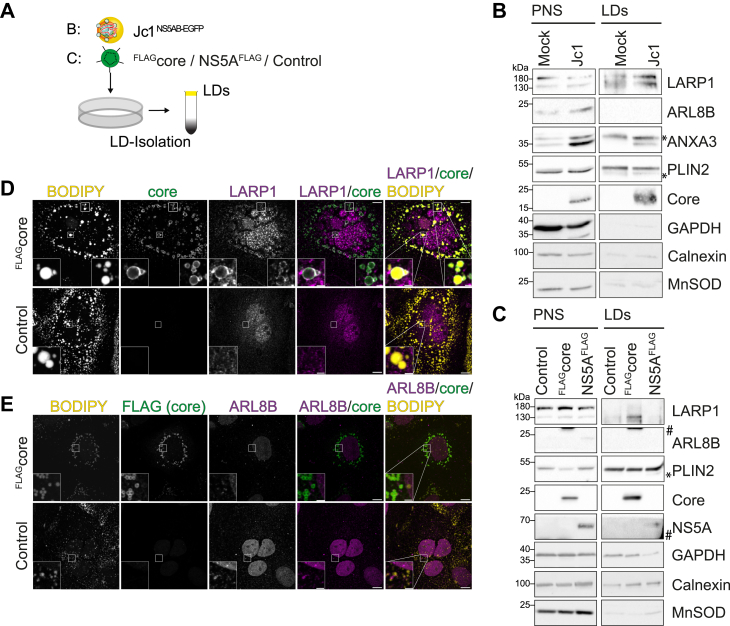
**LARP1 but not ARL8B is recruited to LDs by HCV core.***A*, experimental setup to study LARP1 and ARL8B recruitment to LDs. HCV-infected or lentivirus-transduced cells were lysed and LDs were isolated by sucrose density centrifugation. *B*, Huh7.5 cells were infected with Jc1^NS5AB-EGFP^ (MOI 0.1) and LDs were isolated 14 dpi and analyzed by immunoblotting. *C*, LDs were isolated from Huh7 cells transduced with lentivirus expressing ^FLAG^core, NS5A^FLAG^, or an empty control and analyzed by immunoblotting. *B* and *C*, membranes were probed using specific antibodies against the indicated proteins. GAPDH served as loading control for the post-nuclear supernatant (PNS) and PLIN2 for LD fractions. *Asterisks* mark unspecific bands and # mark bands from previous membrane exposure. Shown are representative experiments (n = 3). *D* and *E*, to study LARP1 (*D*) and ARL8B (*E*) localization in HCV core-expressing cells, Huh7 cells were transfected with expression plasmids for ^FLAG^core. Three days post transfection, cells were fixed and stained with the indicated antibodies. LDs were stained using BODIPY 493/503. *White* squares indicate enlarged areas (n = 2, scale bars represent 10 μm, scale bars in zoom areas represent 2 μm). dpi, days post infection; HCV, hepatitis C virus; LARP1, La-related protein 1; LD, lipid droplet; MOI, multiplicity of infection.

To validate an HCV-dependent LD localization of LARP1 and ARL8B, we isolated LD fractions from Jc1^NS5AB-EGFP^-infected and control Huh7.5 cells by sucrose gradient centrifugation and analyzed them by immunoblotting (Fig. 4*A*). Successful isolation of LDs was confirmed by PLIN2 enrichment, the major LD-coating protein in hepatocytes, and depletion of calnexin and MnSOD as markers for ER and mitochondria, respectively (Fig. 4*B*). Confirming our previous results, ANXA3 levels were increased in LD fractions of HCV-infected compared to uninfected cells (Fig. 4*B*). Despite being proximate to ANXA3 in HCV-infected cells, ARL8B was not detected in LD fractions. We did, however, observe slightly increased ARL8B levels in the post-nuclear supernatant of HCV-infected cells, in agreement with previous reports (21). In contrast, LARP1 was found in increased levels in the LD fractions of HCV-infected cells compared to uninfected controls (Fig. 4*B*). ANXA3 is localized to LDs by the viral proteins core and NS5A (17), which are the two viral proteins that strongly localize to LDs in uninfected cells (13). To investigate if viral protein expression is sufficient to mediate LARP1 recruitment to LDs, we analyzed LD fractions of Huh7 cells stably expressing either ^FLAG^core or NS5A^FLAG^ and found LARP1-enriched in LD fractions of core but not NS5A-expressing cells (Fig. 4*C*). Again, ARL8B was not detectable in LD fractions, but expression levels of ARL8B in uninfected cells were extremely low and only detectable in NS5A^FLAG^-expressing cells.

We then analyzed ^FLAG^core-expressing cells by immunofluorescence microscopy and detected signals of endogenous LARP1 colocalizing with HCV core at LDs (Fig. 4*D*). Confirming the data of the biochemical LD isolations, LARP1 strongly localized to ring-like structures surrounding LDs in core-containing but not in control cells (Fig. 4*D*). Likewise, we investigated the ARL8B localization in ^FLAG^core-expressing cells (Fig. 4*E*). In some areas, ARL8B displayed a punctuate localization in close proximity to LDs but did not colocalize with core at LDs (Fig. 4*E*); however, the signal intensity was very weak.

### LARP1 interacts with HCV core in an RNA-dependent manner

**Figure 5:**
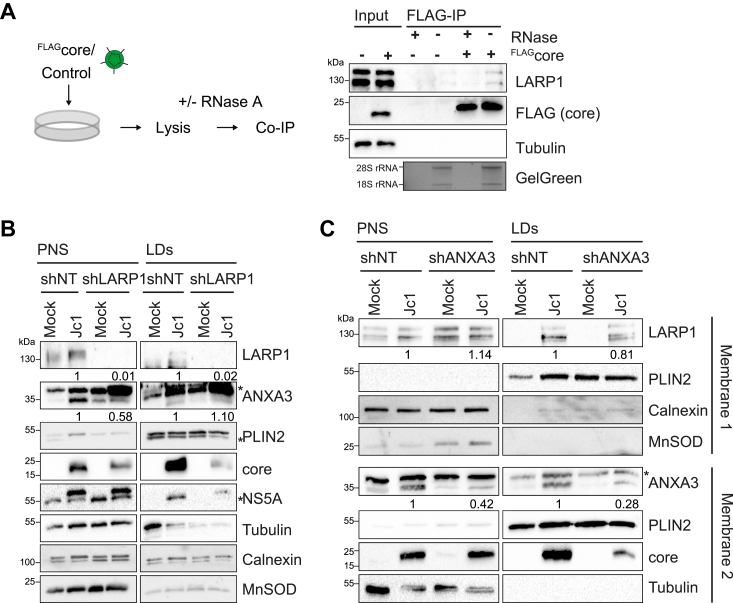
**LARP1 localizes to LDs independently of ANXA3 and interacts with HCV core in an RNA-dependent manner.***A*, Huh7.5 cells were transduced with lentivirus for ^FLAG^core expression or a control. Lysates were pre-treated with RNAse A or RNAseOut and subjected to FLAG-specific immunoprecipitation. Samples were analyzed by immunoblotting using FLAG and LARP1-specific antibodies. Tubulin served as loading control. Successful RNAse A treatment was confirmed by agarose gel electrophoresis and GelGreen staining. Shown is one representative experiment (n = 3). *B*, Huh7 cells transduced with lentivirus carrying shLARP1 or shNT were infected with Jc1^NS5AB-EGFP^. LDs were isolated when all cells were HCV-positive (21 dpi), and post-nuclear supernatants (PNS) and LD fractions were analyzed by immunoblotting. Tubulin served as loading control for PNS samples and PLIN2 for LDs, respectively. Asterisks indicate unspecific bands. Numbers below LARP1 and ANXA3 bands indicate intensity of the respective bands normalized to tubulin for PNS and PLIN2 levels for LD fractions and the shNT control. Shown is one representative experiment (n = 3). *C*, Huh7 cells transduced with lentivirus carrying shANXA3 or shNT were infected with Jc1^NS5AB-EGFP^. LDs were isolated when all cells were HCV-positive (9 dpi), and PNS and LD fractions were analyzed by immunoblotting. Tubulin served as loading control for PNS and PLIN2 for LD fractions. The same samples were loaded on two different gels and probed for the indicated antibodies (membrane 1 and 2). *Asterisks* indicate unspecific bands. Numbers below LARP1 and ANXA3 bands indicate intensity of the respective bands normalized to tubulin for PNS and PLIN2 levels for LD fractions and the shNT control (n = 1). ANXA3, annexin A3; dpi, days post infection; HCV, hepatitis C virus; LARP1, La-related protein 1; LD, lipid droplet.

As we identified LARP1 proximal to LDs upon HCV core expression, we next assessed whether LARP1 directly interacts with HCV core. We confirmed the interaction of core with endogenous LARP1 using Huh7.5 cells transduced with lentivirus-expressing ^FLAG^core (Fig. 5*A*). Since previous reports describe that core binds RNA and interacts with host proteins in an RNA-dependent manner (54, 55, 56) and that LARP1 is part of an RNP complex located at core-containing LDs (42), we treated lysates with RNAse A prior to immunoprecipitation with FLAG beads (Fig. 5*A*). In line with an RNA-dependency, interaction was lost in RNAse A–treated samples, illustrating that LARP1 and core interact in an RNA-dependent manner.

Therefore, LARP1 is part of an HCV core complex that mediates the translocation to LDs. We next wanted to determine which step of the HCV viral life cycle is dependent on LARP1.

### LARP1 and ANXA3 independently localize to LDs in HCV-infected cells

Regarding LD localization of the two host proteins LARP1 and ANXA3, we next analyzed if knockdown of LARP1 would affect LD localization of ANXA3 or vice versa. To deplete ANXA3, we used an shRNA construct described before (17). We constructed lentiviral vectors encoding shRNAs targeting LARP1 and determined the knockdown efficiency by qRT-PCR and immunoblotting (Fig. S2, *A* and *B*). The shRNAs induced a robust knockdown of the respective mRNAs and almost complete depletion on protein level compared to a non-targeting control shRNA (shNT). To rule out cell toxicity or growth defects, we determined the cell viability of cells transduced with the different shRNAs. Cells that harbored LARP1 shRNAs showed a similar viability as control cells (Fig. S2*C*). Cells transduced with shLARP1 or shANXA3 lentiviruses were infected with a Jc1^NS5AB-EGFP^ strain and LD fractions were isolated and analyzed by immunoblotting. LARP1 and ANXA3 are both localizing to LD fractions likely independent of the other protein (Fig. 5, *B* and *C*). ANXA3 was marginally increased at LDs in shLARP1-transduced cells (Fig. 5*B*). And while only ∼30% of ANXA3 was left at LDs in ANXA3-knockdown cells, 80% of LARP1 was found at LDs compared to shNT-transduced cells (Fig. 5*C*). Thus, while they both localize to LDs upon HCV core expression, they do not depend on each other for trafficking to LDs (Fig. 5, *B* and *C*).

### HCV infection depends on LARP1 and ARL8B

**Figure 6:**
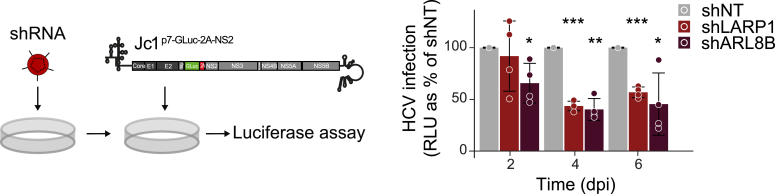
**ARL8B and LARP1 are host factors for HCV infection.** Huh7.5 cells were transduced with lentivirus coding for either shARL8B, shLARP1, or shNT (non-targeting). At 3 dpt, cells were infected with Jc1^p7-GLuc-2A-NS2^ (MOI 0.5) and luciferase activity was measured in the supernatant 2, 4, and 6 dpi. Shown are relative light units (RLU) as percent of shNT (Mean ± SD, n = 4, unpaired two tailed *t* test with unequal variance (Welch’s *t* test) ∗*p* ≤ 0.05, ∗∗*p* ≤ 0.01, ∗∗∗*p* ≤ 0.001). dpi, days post infection; HCV, hepatitis C virus; LARP1, La-related protein 1; MOI, multiplicity of infection.

To confirm a functional role of LARP1 and ARL8B during HCV infection, we infected shRNA-transduced cells with a low multiplicity of infection (MOI) of an HCV *Gaussia* luciferase reporter virus (Jc1^p7-GLuc-2A-NS2^) and viral spread was analyzed 2, 4, and 6 days post infection (dpi) by measuring the *Gaussia* luciferase activity in culture supernatants as a proxy for HCV infection. In line with earlier reports (21, 42, 43), knocking down either ARL8B or LARP1 had a detrimental effect on HCV infection kinetics (Fig. 6). Next, we complemented shLARP1 or shARL8B cells with lentivirus expressing either LARP1 or ARL8B and infected the cells with the Jc1^p7-GLuc-2A-NS2^ reporter virus and analyzed viral spread 2, 4, and 6 dpi (Fig. S3*A*). Overexpression of LARP1 in shLARP1 cells could partially rescue the effect of LARP1 depletion. Notably, LARP1 overexpression alone did not alter viral replication (Fig. S3*A*). We observed no difference in ARL8B-transduced shARL8B cells; however, ARL8B complementation in infected shARL8B cells did not rescue ARL8B protein levels (Fig. S3*B*). These experiments established that HCV spreading depends on LARP1.

### LARP1 knockdown does not affect HCV RNA replication and virion production

**Figure 7:**
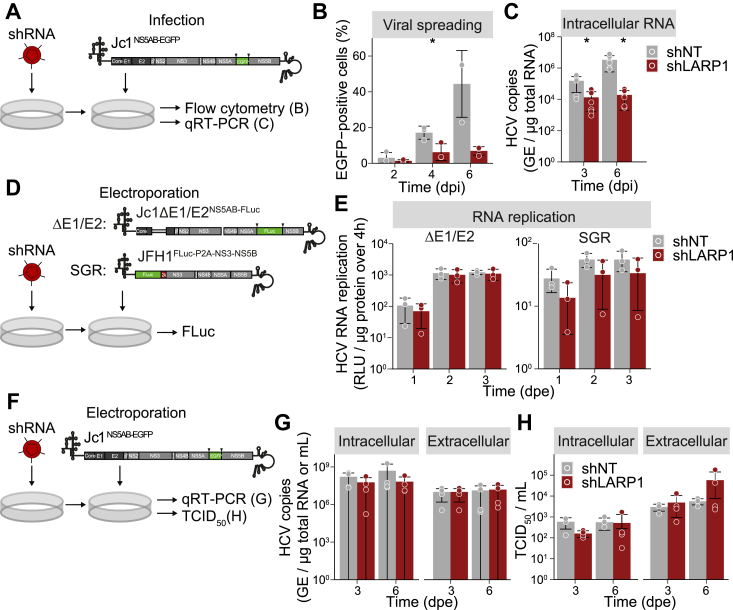
**HCV infection is impaired in LARP1-knockdown cells.***A*, experimental setup to study HCV spreading in LARP1-knockdown cells. shRNA-transduced cells were infected with Jc1^NS5AB-EGFP^ (MOI 0.002) and analyzed by flow cytometry or qRT-PCR at the indicated time points post infection. *B*, shown are relative numbers of EGFP-positive cells (Mean ± SD, n = 3, unpaired two tailed *t* test with unequal variance (Welch’s *t* test) ∗*p* ≤ 0.05). *C*, depicted are HCV RNA copy numbers (GE, genome equivalents) per μg of total RNA (Mean ± SD, n = 3, unpaired two tailed *t* test with unequal variance (Welch’s *t* test) ∗*p* ≤ 0.05). *D*, experimental setup to investigate LARP1-dependent HCV RNA replication. *E*, following transduction, cells were electroporated with Jc1ΔE1/E2^NS5AB-FLuc^ (ΔE1/E2) or JFH1^Fluc-P2A-NS3-NS5B^ (SGR) RNA. HCV RNA replication was analyzed by luciferase assays at 1, 2, and 3 dpe. Shown are RLU per μg protein normalized to the 4 h time point (Mean ± SD, n = 3). *F*, experimental setup to study intracellular and extracellular HCV copy numbers and infectivity from LARP1-depleted or control cells. Transduced cells were electroporated with Jc1^NS5AB-EGFP^ RNA. *G*, three and six dpe, total cellular RNA and viral RNA from supernatant was isolated and HCV RNA copy numbers were determined by qRT-PCR. Shown are the intracellular and extracellular HCV RNA copy numbers per μg of total RNA or per milliliter supernatant (mL), respectively (Mean ± SD, n = 4). *H*, intracellular and extracellular infectious titers were determined by TCID_50_ titration on Huh7.5 RFP–NLS–IPS reporter cells. For intracellular titers, equal numbers of electroporated cells were lysed by freeze-thawing. Shown is the TCID_50_/ml. (Mean ± SD, n = 4). HCV, hepatitis C virus; LARP1, La-related protein 1; MOI, multiplicity of infection; SGR, subgenomic replicon; TCID_50_, 50% tissue culture infective dose.

First, to confirm the data from the luciferase assays, LARP1-knockdown cells were infected with the Jc1^NS5AB-EGFP^ reporter strain, and viral spreading was analyzed by flow cytometry (Fig. 7*A*). The percentage of HCV-positive cells was markedly reduced in LARP1-knockdown cells compared to the control (Fig. 7*B*), indicating that spreading of HCV was severely impaired. In line, HCV RNA levels were significantly reduced in LARP1-knockdown cells 3 and 6 dpi (Fig. 7*C*). These data demonstrate that HCV spreading kinetics are slower in LARP1-knockdown cells.

As others have previously reported an effect of LARP1 on HCV RNA replication (42), we investigated HCV RNA replication by using subgenomic HCV luciferase reporter constructs in LARP1-knockdown cells (Fig. 7*D*). We electroporated shLARP1 or shNT control cells with Jc1ΔE1/E2^NS5AB-FLuc^ RNA and measured the Firefly luciferase activity at different time points post electroporation (dpe). Using our experimental setup, knockdown of LARP1 did not alter HCV RNA replication kinetics (Fig. 7*E*). Considering that HCV core encoded in this replicon construct can redistribute LARP1 to LDs and subsequently might affect HCV replication levels, we additionally performed luciferase assays using a JFH1^FLuc-P2A-NS3-NS5B^ subgenomic replicon (SGR). But again, we did not detect a significant effect on HCV RNA replication in LARP1-knockdown (Fig. 7*E*). Thus, our data suggest that steps of the HCV replication cycle other than RNA replication are more likely to be responsible for the strong reduction of HCV spread observed upon LARP1 knockdown.

Published data concerning LARP1-dependent particle production is conflicting (42, 43) with data suggesting that LARP1 knockdown either impairs or increases HCV particle release. To study infectious virion production in LARP1-knockdown cells, we electroporated shLARP1 or shNT cells with Jc1^NS5AB-EGFP^ RNA. We isolated total cellular RNA as well as viral RNA from the supernatant at 3 and 6 dpe and determined HCV copy numbers by qRT-PCR (Fig. 7*F*). In agreement with our previous data and confirming equal transfection rates, intracellular HCV copy numbers were not affected by LARP1 depletion (Fig. 7*G*). The extracellular HCV copy number, surprisingly, also did not change in LARP1-knockdown cells in comparison to control cells (Fig. 7*G*). As viral RNA levels do not necessarily correlate to viral titers, we measured viral titers by determining the 50% tissue culture infective dose (TCID_50_) (57). LARP1-knockdown and control cells were transfected with Jc1^NS5AB-EGFP^ RNA as described above, and intracellular and extracellular infectious HCV titers were measured in freeze-thaw lysates and culture supernatants, respectively. Depletion of LARP1 did not significantly change intracellular or extracellular infectivity (Fig. 7*H*).

Taken together, our data suggest that LARP1 knockdown does not inhibit HCV RNA replication or HCV particle production but impairs viral spreading.

### Cell-to-cell transmission is impaired in LARP1-deficient cells

**Figure 8:**
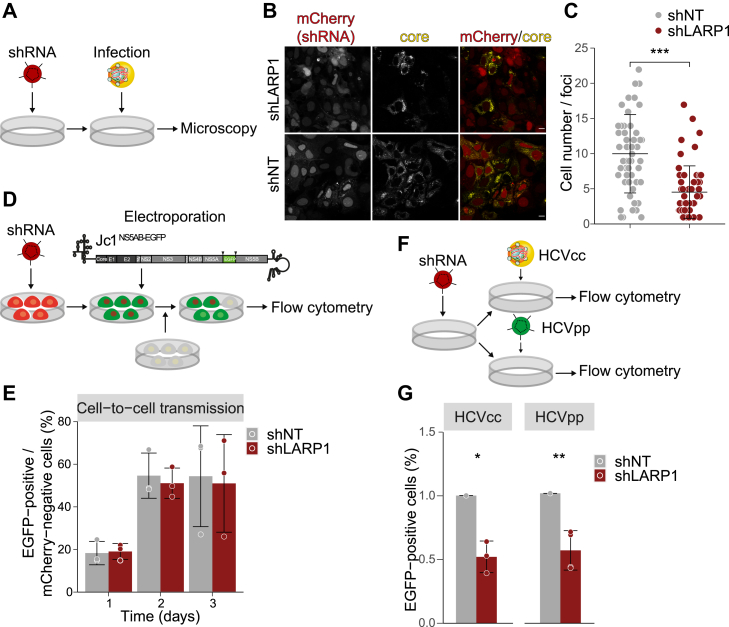
**LARP1 knockdown impairs HCV infection kinetics by interfering with viral entry.***A*, Huh7.5 cells transduced with lentivirus expressing shLARP1 or shNT were infected with Jc1^NS5AB-EGFP^ (MOI 0.1) and fixed 3 dpi. *B* and *C*, for immunofluorescence analysis, cells were stained with a core-specific antibody (*yellow*) and mCherry expression (*red*) is shown as transduction control. The number of infected cells per foci was counted on coverslips from at least two wells in two independent experiments (# of foci: 57 (shNT), 53 (shLARP1); mean ± SD, unpaired two tailed *t* test with unequal variance (Welch’s *t* test) ∗∗∗*p* ≤ 0.001, scale bars represent 10 μm). *D*, scheme for experimental setup to study HCV cell-to-cell transmission. *E*, Huh7.5 cells were transduced with lentivirus expressing shLARP1 or shNT. At 3 dpt, cells were electroporated with Jc1^NS5AB-EGFP^. At 3 dpe, cells were mixed with naïve Huh7.5 cells in an 1:10 ratio and fixed after 1, 2, and 3 days for flow cytometry. Shown are relative numbers of EGFP-positive cells of mCherry-negative cells (Mean ± SD, n = 3). *F*, experimental setup to study HCV entry using HCVcc and HCVpp. Huh7.5 cells were transduced with lentivirus expressing shLARP1 or shNT. *G*, three days after transduction, cells were infected with Jc1^NS5AB-EGFP^ (MOI 0.3) and cells were fixed 24 hpi for flow cytometry (HCVcc) or transduced with HCVpp and analyzed by flow cytometry 3 days later. Shown are relative numbers of EGFP-positive cells (Mean ± SD, HCVcc: n = 3; HCVpp: n = 4, unpaired two tailed *t* test with unequal variance (Welch’s *t* test) ∗*p* ≤ 0.05, ∗∗*p* ≤ 0.01). dpi, days post infection; HCV, hepatitis C virus; LARP1, La-related protein 1; MOI, multiplicity of infection.

Several studies suggest that HCV spreading to neighboring hepatocytes is not only occurring *via* secreted cell-free virions but also *via* direct cell-to-cell transmission (58, 59, 60). As we observed a strong decrease in HCV spreading, we next studied cell-to-cell transmission in LARP1-knockdown cells by determining the number of infected cells per infection foci using immunofluorescence analysis. shLARP1 and shNT cells were infected with HCV at a low MOI and stained with a core-specific antibody 3 dpi (Fig. 8*A*). mCherry expression of the lentiviral shRNA construct confirmed the transduction of the target cells. In line with the earlier experiments, the number of infected cells per foci was significantly reduced in LARP1-deficient cells compared to the control (Fig. 8, *B* and *C*), indicating impaired cell-to-cell transmission of HCV in LARP1-knockdown cells. To further confirm this result using a second assay, we mixed naive cells with a Jc1^NS5AB-EGFP^ electroporated population of LARP1-knockdown or control cells in a 1:10 ratio (Fig. 8*D*). We evaluated the spreading efficiency by analyzing the ratio of EGFP-positive receiver cells 3 days after combining the populations. Unexpectedly, we found that viral transmission from LARP1-depleted cells did not differ from control cells (Fig. 8*E*), arguing for compromised viral entry into cells with reduced LARP1 protein levels rather than impaired virion production.

### LARP1 is a host factor for viral entry

Finally, to analyze the role of LARP1 on HCV entry, we used two different approaches: cell culture–derived HCV particles (HCVcc) and HCV E1E2 pseudotyped lentiviral particles (HCVpp). To study HCVcc entry, we utilized Jc1^NS5AB-EGFP^ virus stock, inoculated shLARP1 or shNT cells with a high MOI, and analyzed them by flow cytometry 24 h post infection (Fig. 8*F*). HCVcc entry was significantly reduced when LARP1 was downregulated in the target cell population (Fig. 8*G*). Similarly, entry of HCVpp was markedly reduced (Fig. 8*G*), indicating that viral entry and not post-entry steps as translation of the viral genome or the formation of replication complexes is dependent on LARP1 as hepacivirus and lentiviruses have differing cellular requirements.

**Figure 9:**
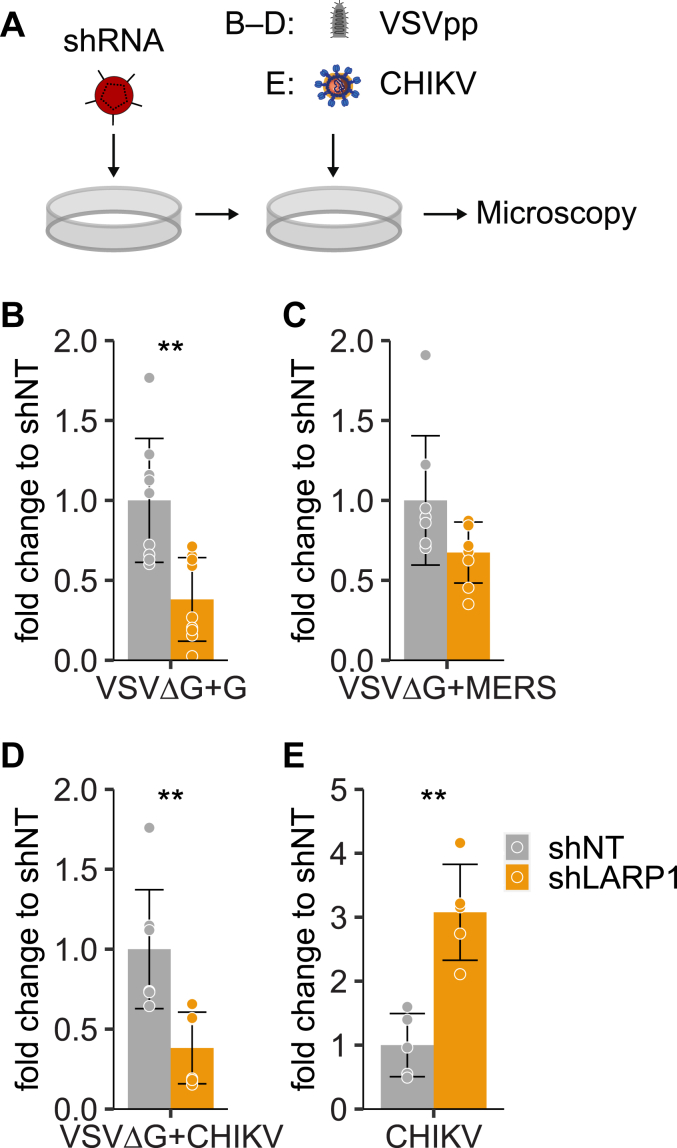
**LARP1 is a general host factor for viral entry.***A*, scheme of the experiment. Huh7 cells transduced with lentivirus expressing shLARP1 or shNT were infected with VSVΔG GFP reporter viruses pseudotyped with the indicated viral glycoproteins (*B*–*D*) or infected with CHIKV-GFP (ECSA stain) (*E*). Infections were analyzed by quantification of the integrated intensity of GFP fluorescence at 12 hpi acquired *via* automated fluorescence microscopy. Shown is fold change over shNT (mean ± SD, n = 9 (VSVΔG+G), n = 8 (VSVΔG + MERS/CHIKV), and n = 5 (CHIKV infection); unpaired two tailed *t* test with unequal variance (Welch’s *t* test) ∗∗*p* ≤ 0.01). CHIKV, chikungunya virus; LARP1, La-related protein 1; MERS, Middle East respiratory syndrome coronavirus; VSV, vesicular stomatitis virus.

To further investigate if LARP1 has a broad effect on virus particle entry, we used a vesicular stomatitis virus (VSV) system with a glycoprotein deletion (VSVΔG) and studied entry of pseudotyped particles displaying glycoproteins of different viruses in LARP1-deficient cells (Fig. 9*A*). Particles pseudotyped with VSV glycoprotein (VSVΔG+G), Middle East respiratory syndrome coronavirus (MERS) glycoprotein (VSVΔG+MERS) as well as chikungunya virus (CHIKV) glycoprotein (VSVΔG+CHIKV) showed decreased entry in shLARP1 cells (Fig. 9, *B*–*D*). Strikingly, infection with full-length CHIKV was significantly increased in LARP1-knockdown cells (Fig. 9*E*), similarly to what has been observed for coronaviruses (61).

Thus, our data collectively suggest that LARP1 acts as a nonspecific pro-viral factor for viral entry, likely by regulating endocytosis.

## Discussion

During HCV infection, the host cell undergoes extensive alterations including membrane rearrangements required for viral RNA replication (8, 62) as well as host protein recruitment to LDs to promote virion morphogenesis (17, 39, 40, 42). We previously demonstrated that HCV infection alters the LD proteome composition and found that the calcium-dependent phospholipid-binding protein ANXA3 is recruited to LDs in HCV-infected cells and plays a crucial role for the incorporation of APOE into viral particles (17). Considering the pivotal role of ANXA3 on HCV particle production at LDs, we used ANXA3 as a bait to further study HCV morphogenesis and release. We performed proximity labeling with the recently described biotin ligase BioID2 (18) in combination with SILAC to identify ANXA3-proximate proteins and to directly compare HCV-infected to uninfected cells. In uninfected hepatoma cells, ANXA3-proximate proteins are cytoskeleton proteins, interactors, or regulators. Of note, ANXA3 is not a well-studied protein on a molecular level and the identified proteins may be a starting point for future characterization of ANXA3 function. However, the cellular compartments identified have been described to be relevant for other annexin family members (47, 48, 49, 50, 52). Out of the 13 significantly enriched proximal proteins in HCV-infected ANXA3-BioID2–expressing cells compared to uninfected cells, we investigated LARP1 and ARL8B in more detail as both had been implicated as host factors involved in HCV assembly (21, 42, 43). In line with what others have shown before, we observed reduced HCV spreading in ARL8B-knockdown cells but no effect on HCV RNA replication (21). HCV infection caused elevated ARL8B protein levels. However, we were not able to detect ARL8B in LD fractions of HCV-infected cells or surrounding LDs during HCV infection. Therefore, the increased detection of ARL8B in close proximity to ANXA3 in HCV-infected cells likely reflects the increased protein levels. Others have shown that ARL8B facilitates lipid mobilization from LDs to lysosomes though membrane contacts sides (22). As mentioned before, the cell undergoes major lipid rearrangements during HCV infection, and it is likely that due to increased lipid turnover, ARL8B can be detected in elevated amounts close to LDs during HCV infection.

The second highly enriched protein we studied in detail was LARP1. LARP1 has a pro-viral effect in dengue virus (37) and HCV (42, 43). LARP1-deficient cells did not efficiently support HCV spreading infection. This corresponds well with previously published data (42). However, we only observed a marginal reduction in HCV RNA replication levels when LARP1-knockdown cells were electroporated with two different replicon systems in contrast to the strong negative effect on HCV RNA replication following LARP1 knockdown in a stable SGR cell line shown before (42). Contrary to what others have reported before, we did not observe any effect of LARP1 on HCV virion production. This step was previously demonstrated to be either impaired or increased by LARP1 knockdown (42, 43).

Our data clearly suggest that LARP1 is required for efficient HCV spreading at early steps in the viral life cycle without altering HCV RNA replication or the formation and secretion of infectious particles. Immunofluorescence analysis of Jc1-infected LARP1-knockdown cells revealed significantly smaller foci of infected cells than in control cells. We demonstrate that the detrimental effect of LARP1 knockdown on HCV spread is due to defects in viral entry into LARP1-deficient cells rather than defects in the HCV producer cells. These findings uncouple the relocalization of LARP1 to HCV assembly sites from its function in HCV infection and point to a broader role of LARP1 in regulating viral entry.

Interestingly, in HeLa cells, expression of LARP1 affects cytoskeleton organization. In LARP1 RNAi-treated cells, actin diffusely localizes in the cytoplasm, whereas LARP1 overexpression leads to a redistribution of γ-actin towards the cell membrane within lamellipodia (36), which may affect endocytosis. Recent studies also showed that mTOR inhibition regulated the RNA-binding specificity as well as translational functions of LARP1 (63, 64). Further, inhibition of mTOR regulates the degradation of interferon-induced transmembrane proteins (65). These proteins inhibit virus-cell fusion for various viral infections (66, 67). Using the recombinant VSV system in combination with glycoproteins from different viruses, including MERS and CHIKV, we could show a broader dependency of viral entry on LARP1 expression. Interestingly, full-length infection with CHIKV increased viral replication in the absence of LARP1. Similar results have been shown for SARS-CoV-2 replication (61). Viral RNA levels and infectious virus production were significantly elevated in LARP1-depleted cells. The same study identified that LARP1 binds SARS-CoV-2 RNA, predominantly 5′UTR sequences and TOP-like sequences, suggesting that LARP1 binds these 5′TOP motifs to regulate translation and suppress viral replication. Notably, LARP1 was identified to bind incoming pre-replicated CHIKV RNA (68). Therefore, LARP1 could bind CHIKV TOP-like sequences in a similar manner. HCV translation is mediated by an internal ribosomal binding site (IRES). Of note, the unusual flavin adenine dinucleotide cap in HCV protects the RNA from cell-intrinsic innate immune recognition (69). Thus, it is likely that LARP1 is not directly binding HCV RNA but rather triggers the hosts’ translation machinery leading to an opposite effect compared to viruses with a cap. Taken together, our data expand the role of LARP1 as a pro-viral factor for HCV entry, likely by regulating the global translatome that controls viral endocytosis.

## Experimental procedures

### Cell lines, culture conditions, and viability assays

Huh7.5 cells were provided by Charles M. Rice, and HEK293T cells were obtained from the American Type Culture Collection. Huh7 cells were from Ralf Bartenschlager and Huh7.5.1 were obtained from Apath, LLC. Cells were grown in Dulbecco’s modified Eagle’s medium (DMEM, high glucose) supplemented with 10% fetal calf serum, 1% glutamine, and 1% penicillin/streptomycin under standard cell culture conditions. All cell lines were authenticated by STR fingerprinting and were regularly tested for *mycoplasma* contamination. Cell viability was determined by CellTiter96 Aqueous One Solution Reagent (Promega). Transfection was performed either using Fugene 6 (Promega) or calcium phosphate precipitation method.

### Antibodies and reagents

The following antibodies, dyes, beads, and chemicals were commercially purchased: LARP1 (NBP1-19128, Novus Biologicals, 1:1000 WB, 1:50 IF), HCV core (clone 7-50, sc-57800, Santa Cruz Biotechnology, 1:250 WB, 1:20 IF), PLIN2 (610102 Progen, 1:250 WB), tubulin (T6074, Sigma, 1:2000 WB), HCV NS5A (HCM-131-5, IBT, 1:250 WB, 1:20 IF; MAB8694, Merck, 1:250 WB), HCV NS3 (ab65407 Abcam, 1:1000 WB), FLAG (F7425, Sigma, 1:1000 WB; F1804, Sigma, 1:250 WB), ANXA3 (hpa013431, Sigma, 1:1000 WB), ARL8A/B (sc-398679, Santa Cruz Biotechnology, 1:250 WB, 1:20 IF), GAPDH (sc-365062, Santa Cruz Biotechnology, 1:250 WB), calnexin (sc6465, Santa Cruz Biotechnology, 1:250 WB), MnSOD (ADI-SOD-111, Enzo, 1:1000 WB), streptavidin agarose resins (20349, Thermo Fisher Scientific), anti-FLAG M2 affinity gel (A2220, Sigma), recombinant protein G agarose beads (15920-010, Sigma), HRP-labeled secondary antibodies (Jackson ImmunoResearch, 1:10000 WB), HRP-labeled TrueBlot secondary antibodies (Rockland Immunochemicals), Alexa 488-, Alexa 647-conjugated secondary antibodies (donkey, IgG (H+L), Life Technologies, 1:1500 IF), BODIPY 493/503 (D-3922, Life Technologies, 1:750 IF), BODIPY 655/676 (B3932, Life Technologies, 1:20000 IF), Hoechst33342 (Thermo Fisher Scientific, 1:6000 IF), oleic acid (O3008-5 ml, Sigma), biotin (B4501, Sigma), Coelenterazine (Carl Roth) Roti-Blue Coomassie Staining (A162.2, Carl Roth), Pierce 660 nm Protein Assay (Thermo Fisher Scientific), DC Protein Assay (Bio-Rad), Pierce Coomassie Plus Bradford Assay (Thermo Fisher Scientific). Specificity of the ANXA3 and PLIN2 antibodies has been confirmed in previous publications in ANXA3-knockdown as well as knockout cells and PLIN2-knockdown cells, respectively (14, 17).

Oligonucleotides and PCR primers were obtained from Sigma. Restriction enzymes for molecular cloning were purchased from NEB. If not noted otherwise, chemicals were generally purchased from AppliChem, Sigma, or Merck.

### Plasmids and HCV constructs

The proximity labeling construct MCS-BioID2-HA was a gift from Kyle Roux (Addgene plasmid # 74224). ANXA3-BioID2^HA-2A-mCherry^ or BioID2^HA-2A-mCherry^ were cloned into the lentiviral pSicoR-MS1 lacking the U6 promotor by two steps of overlap extension PCR using a pEBB-ANXA3-HA expression plasmid (17), MCS-BioID2-HA, and pSicoR-MS1 with an mCherry as templates (primers: fw pSicoR ANXA3: CTGTGACCGGCGCCTACGATGGCATCTATCTGGGTT; rev ANXA3-BioID2-HA: CAGGTTCTTGAACATACCACCGTCATCTCC; fw ANXA3-BioID2-HA: GATGACGGTGGTATGTTCAAGAACCTGATC; rev pSicoR BioID2-HA: TAGGTCCCTCGACGAATTTTATGCGTAATCCGGTAC; rev BioID2-HA-2A: GACGTCTCCCGCAAGCTTAAGAAGGTCAAAATTTGCGTAATCCGGTAC; fw NheI BioID2: GCCTACGCTAGCATGTTCAAGAACCTGATC; fw 2A-mCherry: TGCGGGAGACGTCGAGTCCAACCCTGGGCCAGTGAGCAAGGGCGAG; rev EcoRI-mCherry: CTCGACGAATTCTTACTTGTACAG) at NheI and EcoRI restriction sites.

The lentiviral shRNA constructs targeting LARP1 and ARL8B were cloned into pSicoR-MS1 (mCherry) as previously described (12) (shLARP1: GCGCCAGATTGAATACTACTT (42); shARL8B: GCCTGCTTTATCTAATGTAAT). Lentiviral shRNAs targeting ANXA3 and shNT control were described before (17).

For LARP1 and ARL8B overexpression constructs, the multiple cloning site (MCS) from lentiviral pSicoR-MS1 lacking the U6 promotor (17) was removed by restriction digestion with enzymes XbaI and NotI, ends were blunted using T4 DNA polymerase (1 U, NEB) and re-ligated. A new MCS, followed by an IRES-driven EGFP was inserted by PCR (primers: fw NheI-MCS-IRES: GCTAGCTCTAGAACGCGCGGTGACCCTCGAGTACTAGGATCCATTAGGGGATCCGCCCCTCTC; rev EcoRI-EGFP: AGGTCCCTCGACGAATTCTTACTTGTACAGCTCGTCCAT) using an HCV core-IRES-EGFP expression plasmid (12) as a template. The resulting construct was used for further cloning and as an empty vector control in LARP1 and ARL8B overexpression experiments. T7-tagged LARP1 or ARL8B was amplified by PCR (primers: fw SpeI-T7-LARP1: ACCGGCGCCTACACTAGTGCTATGGCTAGCATGACTGGTGGACAGCAAATGGGTATGCTTTGGAGGGTG; rev LARP1-XhoI: GTACTCGAGCTTTCCCAAAGTCTGTGT; fw SpeI-T7-ARL8B: ACCGGCGCCTACACTAGTGCTATGGCTAGCATGACTGGTGGACAGCAAATGGGTATGCTGGCGCTCATCTCCCGC; rev ARL8B-XhoI: GTACTCGAGGCTTCTTCTAGATTT) and inserted by NheI/SpeI and XhoI restriction sites. As a template for the LARP1 or ARL8B, PCR expression plasmids purchased from GenScript (LARP1 Clone ID: C53021; vector: pcDNA3.1+/C-(K)-DYK; ARL8B Clone ID: IP6341; vector: pcDNA3.1+/C-(K)-DYK) was used.

We used the following previously described plasmids: Full length or envelope-deleted HCV Jc1 reporter strains encoding fluorescent proteins and selection markers or a firefly luciferase between a duplicated NS5A-NS5B cleavage site (Jc1^NS5AB-EGFP^, Jc1ΔE1/E2^NS5AB-EGFP-BSD^, Jc1ΔE1/E2^NS5AB-FLuc^) (53), Con1 SGR (70), the secreted *gaussia* luciferase reporter (Jc1^p7-GLuc-2A-NS2^) (17), LeGO-iCer2 lentiviral vectors encoding FLAG-tagged HCV JFH1 core or NS5A (17), a FLAG-tagged HCV core (gt2A) expression plasmid (71), the lentiviral HCV RFP NLS-IPS expression construct (72). The JFH1 SGR with a firefly luciferase (JFH1^FLuc-P2A-NS3-NS5B^) was conducted by overlap PCR using a Jc1 Luc reporter construct backbone (73) as template (primers: fw core-FLuc-p2A: ATG AGC ACA AAT CCT AAA CCT CAA AGA; rev core-FLuc-p2A: ATC ACC TGC TTG CTT TAG CAG AGA GAA GTT TGT GGC GCC GCT GCC CAA TTT GGA CTT TCC GCC; fw p2A-NS3-NS5A: TCT CTG CTA AAG CAA GCA GGT GAT GTT GAA GAA AAC CCC GGG CCT GCT CCC ATC ACT GCT TAT; rev NS3-NS5A: GCA GCA CAC GGT GGT ATC GTC CTC CTC). The resulting PCR product was inserted into the Jc1 Luc reporter construct by restriction digestion with enzymes XbaI and RsrII.

For EGFP-expression used for HCVpp experiments, we used a pSicoR-MS1ΔU6 lentiviral construct carrying an EGFP. A pSicoR-MS1ΔU6 (17) was cut by restriction digestion using NheI and EcoRI followed by insertion of EGFP by ligase independent cloning using a pEGFP-C1 (Clontech) as a template (primers: fw NheI EGFP: GTGACCGGCGCCTACGCTAGATGGTGAGCAAGGGCGAG; rev EGFP EcoRI: ATTAGGTCCCTCGACGAATTTTACTTGTACAGCTCGTC).

The HCVpp envelope plasmid pcDNA3 ΔcE1E2 (JFH1) has been described (74).

### HCV RNA *in vitro* transcription and HCV virus stock production

HCV viral stocks were produced by electroporation of 4∗10^6^ Huh7.5.1 or Huh7.5 cells with 10 μg *in vitro*–transcribed RNA as described before (12, 17). Viral titers were determined by TCID_50_.

### Determination of viral titers (TCID_50_)

Viral titers were determined with the tissue culture infectious dose TCID_50_ using Huh7.5 cells stably expressing the HCV reporter RFP-NLS-IPS (72) as described before and calculated with the Reed and Muench calculator (17, 57). For analysis of intracellular titers, HCV RNA-electroporated cells were trypsinized, and 2 × 10^5^ cells were resuspended in 2 ml DMEM and lysed by multiple freeze/thaw cycles. Cell debris was removed by centrifugation for 5 min at 100*g*, and TCID_50_/ml was determined.

### HCV infection, replicon, and luciferase assays

For HCV spreading experiments, lentiviral-transduced cells were analyzed using flow cytometry or *Gaussia* luciferase assays at the indicated timepoints as described before (17, 75). Cells were either infected with Jc1^NS5AB-EGFP^ (53) and analyzed by flow cytometry with a BD LSR Fortessa (BD Bioscience) and analyzed with FlowJo (Treestar) or infected with Jc1^p7-GLuc-2A-NS2^ (17), and *Gaussia* luciferase activity in the supernatant was measured using Coelenterazine (Carl Roth) and a Centro LB 960 luminometer (Berthold Technologies).

To measure HCV RNA replication, lentiviral-transduced cells were electroporated with Jc1ΔE1/E2^NS5AB-FLuc^ (53) as described in (17) or JFH1^FLuc-P2A-NS3-NS5B^ RNA, and Firefly luciferase activity in cell lysates was determined using Luciferase Assay System (Promega). Protein levels were determined by Coomassie-Plus Assay (Thermo Fisher Scientific).

### Proximity labeling and SILAC labeling for mass spectrometric proteomics

For isotope metabolic protein labeling, we cultured cells in Dundee Cell Products DMEM media (“light”, R0K0; “heavy”, R10K8), supplemented with 10% fetal bovine serum (Biochrom Superior) and 1% Penicillin/Streptomycin (Sigma). Incorporation efficiency after eight passages was determined by mass spectrometric proteomics using cell lysates separated by SDS-PAGE (Mini Protean Gel Any kDa, 456-9034, Bio-Rad). Gels were stained with Roti-Blue dye (Carl Roth) following manufacturer’s instructions and analyzed by mass spectrometric proteomics.

For proximity labeling analysis, Huh7.5 cells were cultivated in light or heavy media for four passages prior to lentiviral transduction of the ANXA3-BioID2^HA-2A-mCherry^ fusion construct or the BioID2^HA-2A-mCherry^ construct. One week after transduction, cells were either directly biotin-labeled or infected with a Jc1^NS5AB-EGFP^ reporter strain (53) and cultured for two more weeks.

For BioID2 labeling, the media was supplemented with 10 μM biotin (Sigma). After 20 h of incubation, cells were washed 5x in PBS and lysed in RIPA buffer (150 mM NaCl, 50 mM Tris–HCl pH 7.6, 1% NP-40, 0.5% sodium deoxycholate, 5 mM EDTA, supplemented with 1x protease inhibitor cocktail (Sigma), and 1 mM PMSF). Protein levels were determined with Pierce 660 nm Protein Assay (Thermo Fisher Scientific) following manufacturer’s instruction. Prior to pull down, 35 μl streptavidin agarose resins (Thermo Fisher Scientific) were washed 3x in RIPA buffer at 425*g* for 2 min, 4 °C for equilibration. Resins were incubated with a total protein amount of 2 mg (1 mg “light” and 1 mg “heavy” labeled sample) for 1.5 to 2 h at 4 °C, rotating. Beads were eluted in Laemmli, supplemented with 2 mM biotin and 20 mM DTT (Thermo Fisher Scientific), and boiled at 95 °C for 10 min. Samples were separated by SDS-PAGE as mentioned above and in-gel digestion was performed as described (76).

### Mass spectrometric proteomics: protein digestion in the SDS-PAGE matrix

Shrinking and swelling was performed with 100% acetonitrile (ACN) and 100 mM ammonium hydrogen carbonate (NH_4_HCO_3_). In-gel reduction was achieved with 10 mM DTT (dissolved in 100 mM NH_4_HCO_3_). Alkylation was performed with 55 mM iodoacetamide (dissolved in 100 mM NH_4_HCO_3_). Proteins in the gel pieces were digested by covering them with a trypsin solution (8 ng/μl sequencing-grade trypsin, dissolved in 50 mM NH_4_HCO_3_) and incubating the mixture at 37 °C overnight. Tryptic peptides were yielded by extraction with 0.1% formic acid (FA) in 80% ACN. Samples were lyophilized using a vacuum centrifuge.

### Mass spectrometric proteomics: LC-MS/MS in data-dependent mode

Samples were resuspended in 0.1% FA and transferred into a full recovery autosampler vial (Waters). Chromatographic separation was achieved on a UPLC system (nanoAcquity, Waters) with a two buffer system (buffer A: 0.1% FA in water, buffer B: 0.1% FA in ACN). Attached to the UPLC was a C18 trap column (Symmetry C18 Trap Column, 100 Å, 5 μm, 180 μm × 20 mm, Waters) for online desalting and sample purification followed by an C18 separation column (BEH130 C18 column, 75 μm × 25 cm, 130 Å pore size, 1.7 μm particle size, Waters). Peptides were separated using a 60 min gradient with increasing ACN concentration from 2 to 30% ACN. The eluting peptides were analyzed on a quadrupole orbitrap mass spectrometer (QExactive, Thermo Fisher Scientific) in data dependent acquisition mode.

For LC-MS/MS analysis on the QExactive, the 15 most intense ions per precursor scan (1 × 10^6^ ions, 70,000 Resolution, 100 ms fill time) were analyzed by MS/MS (HCD at 25 normalized collision energy, 2 × 10^5^ ions, 17,500 Resolution, 50 ms fill time) in a range of 400 to 1200 m/z. A dynamic precursor exclusion of 20 s was used.

### Mass spectrometric proteomics: data analysis and processing

Acquired data dependent acquisition LC-MS/MS data to generate a reference peptide spectra library for SILAC-based MS1 area data extraction were searched against the reviewed human protein data base downloaded from Uniprot (November 2017, as well as protein sequences of proteins introduced by transfection) using the Sequest algorithm integrated in the Proteome Discoverer software version 2.0 (Thermo Fisher). Mass tolerances for precursors were set to 10 ppm and 0.02 Da for fragments. Carbamidomethylation was set as a fixed modification for sulfhydryl residues of cysteine, and ^13^C_6_,^15^N_2_-labeled lysine, ^13^C_6_,^15^N_4_-labeled arginine, the oxidation of methionine, pyro-glutamate formation at glutamine residues at the peptide N terminus as well as acetylation of the protein N terminus, methionine loss at the protein N terminus, and the acetylation after methionine loss at the protein N-terminus were allowed as variable modifications. Only peptide with a high confidence (false discovery rate < 1% using a decoy data base approach) were accepted as identified.

Proteome Discoverer search results were imported into Skyline software (www.skyline.ms) (MacCoss Lab, University of Washington) allowing only high confidence peptides to be imported. Peptide areas for the light and heavy variant of each peptide were extracted and filtered for each sample condition for peptides with an isotope dot product of >0.85 in either light or heavy or in both variants of a given peptide. Peptide peak areas ratios for light over heavy were calculated and a median ratio per protein was calculated which were then used for relative abundance comparison.

### Lentivirus production

Lentiviral particles were produced using HEK293T cells as described before (17, 77). Transduction was performed with cell culture media supplemented with 4 μg/ml polybrene. Titration was done on Huh7.5 cells.

### Immunofluorescence and confocal microscopy

Cells were grown on coverslips and fixed in 4% paraformaldehyde (PFA) for 1 h, washed in PBS, and permeabilized in 0.1% Triton X-100 for 5 min. After blocking (5% bovine serum albumin, 1% fish gelatin, 50 mM Tris in PBS) for 1 h, cells were incubated with primary antibodies diluted in blocking solution o/n at 4 °C. Cells were washed and incubated with Alexa-Fluor fluorescence-coupled secondary antibodies (Life Technologies) diluted in blocking solution (1:1000–1:1500). LDs were stained with BODIPY 493/503 (1:750 in PBS, Life Technologies) or BODIPY 655/676 (1:20,000 in PBS) for 30 min. Coverslips were embedded in mowiol mounting media (78). Confocal microscopy was performed on a Leica Stellaris 8 confocal laser scanning microscope. Further analysis was performed with Fiji (79).

### Co-immunoprecipitation

For immunoprecipitation, cells were lysed in NP40 lysis buffer (50 mM Tris, pH 7.4, 150 mM NaCl, 1% Nonidet-P40, supplemented with 1 mM PMSF, and 1x protease inhibitor cocktail (Sigma)), and protein levels were determined by DC Protein Assay. Lysates were precleared with protein G agarose beads (Sigma) for 30 to 60 min at 4 °C, rotating. To analyze RNA-mediated interactions, lysates were incubated with 100 μg/ml RNAse A (Thermo Fisher Scientific) or 100 U/ml RNAseOUT (Invitrogen) for 45 to 60 min at 4 °C, rotating. Successful RNAse A treatment was confirmed by agarose gel electrophoreses of RNA isolated from lysates using Tri Reagent (Sigma). For immunoprecipitation of tagged proteins, lysates were incubated with anti-FLAG M2 (Sigma) over night at 4 °C, rotating. Beads were washed four times in cold NP40 lysis buffer, eluted in 2x Laemmli buffer, and analyzed by immunoblotting as mentioned above.

### Immunoblot analysis

Cells were lysed 30 min–1 h on ice in RIPA buffer (150 mM NaCl, 50 mM Tris–HCl pH 7.6, 1% NP-40, 0.5% sodium deoxycholate, 5 mM EDTA, supplemented with 1x protease inhibitor cocktail (Sigma), and 1 mM PMSF) or in NP40 lysis buffer (50 mM Tris, pH 7.4, 150 mM NaCl, 1% Nonidet-P40, supplemented with 1 mM PMSF, and 1x protease inhibitor cocktail (Sigma)) for co-immunoprecipitation analysis. Cell debris and nuclei were removed by centrifugation for 10 min at 20,000*g*, 4 °C. Protein levels were determined by DC Protein Assay (Bio-Rad). After SDS-PAGE and blotting on nitrocellulose membranes (GE Healthcare), we used Lumi-Light Western Blotting Substrate (Roche) or SuperSignal West Femto (Thermo Fisher Scientific) and either ECL Hyperfilm (Amersham) or Image Lab (Biorad) for chemiluminescent detection of bands. Signal intensities were quantified using the quantification function of Image Lab (Bio-Rad).

### LD isolation

LD isolations were performed as described before (9, 17). Briefly, cells were scraped in cold PBS, lysed in hypotonic buffer (50 mM Hepes, 1 mM EDTA, 2 mM MgCl_2_, pH 7.4, supplemented with 1x protease inhibitor cocktail (Sigma)) using a Dounce homogenizer for 5 min. Nuclear fractions were removed by centrifugation for 5 min at 500*g*. Postnuclear fractions were mixed with an equal volume of 1.05 M sucrose in isotonic buffer (50 mM Hepes, 100 mM KCl, 2 mM MgCl_2_, supplemented with 1 mM PMSF) in SW60 Ti or SW41 (Beckman) rotor tubes and centrifuged for 2 h at 100,000*g*, 4 °C. The floating fractions were harvested using a bended cannula and centrifuged for 20 min at 20,000*g*, 4 °C. Underlying buffer was removed and to concentrate LD fractions. Triton X-100 was added to all samples from infection experiments to a final concentration of 1% for virus inactivation. Protein levels were determined using DC Protein Assay (Bio-Rad).

### RNA isolation and quantitative RT-PCR

Total cellular RNA was isolated from cells or immunoprecipitation samples using Tri Reagent (Sigma) and RNA was treated with rDNAseI (DNA-free Kit, Invitrogen). Viral RNA from supernatants were isolated using NucleoSpin RNA Virus Kit (Machery Nagel). RNA was reversely transcribed with Superscript III reverse transcriptase (Invitrogen), random hexamer primers (Qiagen), and RNAseOut (Invitrogen). For qRT-PCR analysis, we used Maxima SYBR Green Mastermix (Thermo Fisher Scientific) or Luna Universal qPCR Mastermix (NEB) on a 7500HT Fast Realtime PCR System or a StepOne Plus Real Time qPCR System (Applied Biosystems). qRT-PCR primers were selected from the Harvard primer bank (80). Used qRT-PCR primers: LARP1 fw: GCCTGGCAACCAGAGATCAAA; LARP1 rev: TCAAACTTTCGGTAGCCAAACT; ARL8B fw: CATCGCGTCAGGTCAATTCAG; ARL8B rev: GTTGTCCTCCTATGTCCCAGA; HCV fw: CGGGAGAGCCATAGTGG; HCV rev: AGTACCACAAGGCCTTTCG (12); 18s rRNA fw: GTAACCCGTTGAACCCCATT; 18s rRNA rev: CCATCCAATCGGTAGTAGCG.

### Cell-to-cell transmission analysis

To determine foci size, Huh7.5 cells were transduced with lentivirus carrying shLARP1 or shNT and infected with Jc1^p7-GLuc-2A-NS2^ or Jc1^NS5AB-EGFP^ (MOI 0.1–0.15). Three dpi, cells were fixed in 4% PFA for 1 h at 4 °C and stained for immunofluorescence using a core-specific antibody.

For cell-to-cell transmission assays, Jc1^NS5AB-EGFP^ -electroporated shLARP1 or shNT cells were co-cultured with naïve Huh7.5 cells. Huh7.5 cells were transduced with lentivirus carrying shLARP1 or shNT and electroporated with Jc1^NS5AB-EGFP^. Three days later, cells were mixed with naïve Huh7.5 cells in a 1:10 ratio and cells were fixed for flow cytometry in 2% PFA at the indicated timepoints.

### HCVpp entry assay

HCVpp entry assays were performed as described before (75). Briefly, pseudoparticles were produced with pSicoR-MS1ΔU6 (EGFP reporter), a pCMVdR8.91 packaging construct (77), and pcDNA3 ΔcE1/E2 (JFH1) (74). Huh7.5 cells were transduced with shNT or shLARP1 3 days prior to transduction with HCVpp. Three days later, cells were harvested and fixed for flow cytometry using a Guava EasyCyte and analyzed using FlowJo.

### VSVΔG entry assay

Three days after transduction with shLARP1- or shNT-carrying lentiviral particles, Huh7 cells were transduced with VSV-GFP-FLuc-ΔG (81) pseudotyped with either CHIKV glycoproteins E1-E2 (pIRES2-eGFP CHIKV E3-E1 (82)), MERS Spike (pCAGGS-MERS-S (83)), or VSV-G (pczVSV-G (84)) at a concentration resulting in 10 to 20% infection. Pseudoparticle transduction was measured as integrated intensity of GFP expression at 12 h post infection using the Incucyte S3/SX1 imaging platform (Sartorius). Results of nine experiments with VSVΔG+G and eight experiments with VSVΔG+MERS/CHIKV were normalized to the mean of all shNT-treated samples for statistical analysis.

### CHIKV infection

Three days post transduction with shNT- or shLARP1-expressing lentiviral particles, Huh7 cells were infected with CHIKV encoding GFP (CHIKV-LR2006 OPY1 5′GFP (85)) at an MOI of 0.05. Infection with CHIKV was quantified at 12 hpi using the Incucyte S3/SX1 imaging platform (Sartorius). Integrated GFP intensities measured in five experiments were normalized to the mean of all shNT controls.

### Statistical analysis and bioinformatic analysis of the proteomics results

The detection ratios of light over heavy (L/H) or heavy over light (H/L) were centered by dividing through the median. Analysis was performed using RStudio (86). For plotting, gdata (87), lattice (88), gplots (89), ggplots2 (90), and pheatmap (91) packages were used. GO annotation was generated using the online tool ShinyGO (92) using the sources: GO cellular components resources, KEGG pathways, biological processes, or molecular function with enriched proteins (log_2_ fold change >0.5) with a *p*-value (FDR) cutoff of 0.05 (2023/01/04 and 2023/10/31) (92).

For statistical analysis, R and RStudio was used (86). Analysis was performed using an unpaired two tailed *t* test with unequal variance (Welch’s *t* test). Indicated sample sizes (n) represent independent experiments, if not stated otherwise.

## Data availability

The mass spectrometry proteomics data have been deposited to the ProteomeXchange Consortium *via* the PRIDE (93) partner repository with the dataset identifier PXD049182. All other data described in this study are contained within the article.

## Supporting information

This article contains supporting information.

## Conflict of interest

The authors declare that they have no conflicts of interest with the contents of this article.
