# Supplementary material for: Proximity labeling of host factor ANXA3 in HCV infection reveals a novel LARP1 function in viral entry
Source: J Biol Chem. 2024 Apr 16;300(5):107286. doi: 10.1016/j.jbc.2024.107286 (PMC11101947; doi:10.1016/j.jbc.2024.107286)
Supplement: Supporting Figures [file mmc1.pdf]

Supporting Information

Proximity labeling of host factor ANXA3 in HCV infection reveals a novel LARP1 function in viral entry

Hanna Bley[1], Christoph Krisp[2], Anja Schöbel[1], Julia Hehner[1], Laura Schneider[1], Miriam Becker[3], Cora Stegmann[3], Elisa Heidenfels[1], Van Nguyen-Dinh[1], Hartmut Schlüter[2], Gisa Gerold[3,4,5], and Eva Herker[1]*

[1] Institute of Virology, Philipps-University Marburg, Marburg, Germany,

[2] Section Mass Spectrometry and Proteomics, University Medical Center Hamburg-Eppendorf, Hamburg, Germany

[3] Institute for Biochemistry & Research Center for Emerging Infections and Zoonoses (RIZ), University of Veterinary Medicine Hanover, Hanover, Germany

[4] Department of Clinical Microbiology, Virology, Umeå University, Umeå, Sweden

[5] Wallenberg Centre for Molecular Medicine (WCMM), Umeå University, Umeå, Sweden

Short Title: Novel function of LARP1 in virus entry

* Corresponding author

E-mail: eva.herker@uni-marburg.de.

Supporting Information includes:

Figure S1: Identification of BioID2-proximal proteins in HCV-infected cells

Figure S2: Validation of LARP1 and ARL8B shRNA

Figggure S3: LARP1 overexpression partially rescues its effect on HCV replication in shLARP1 cells.

Table S1: Proteins identified through proximity labeling of ANXA3-BioID2.

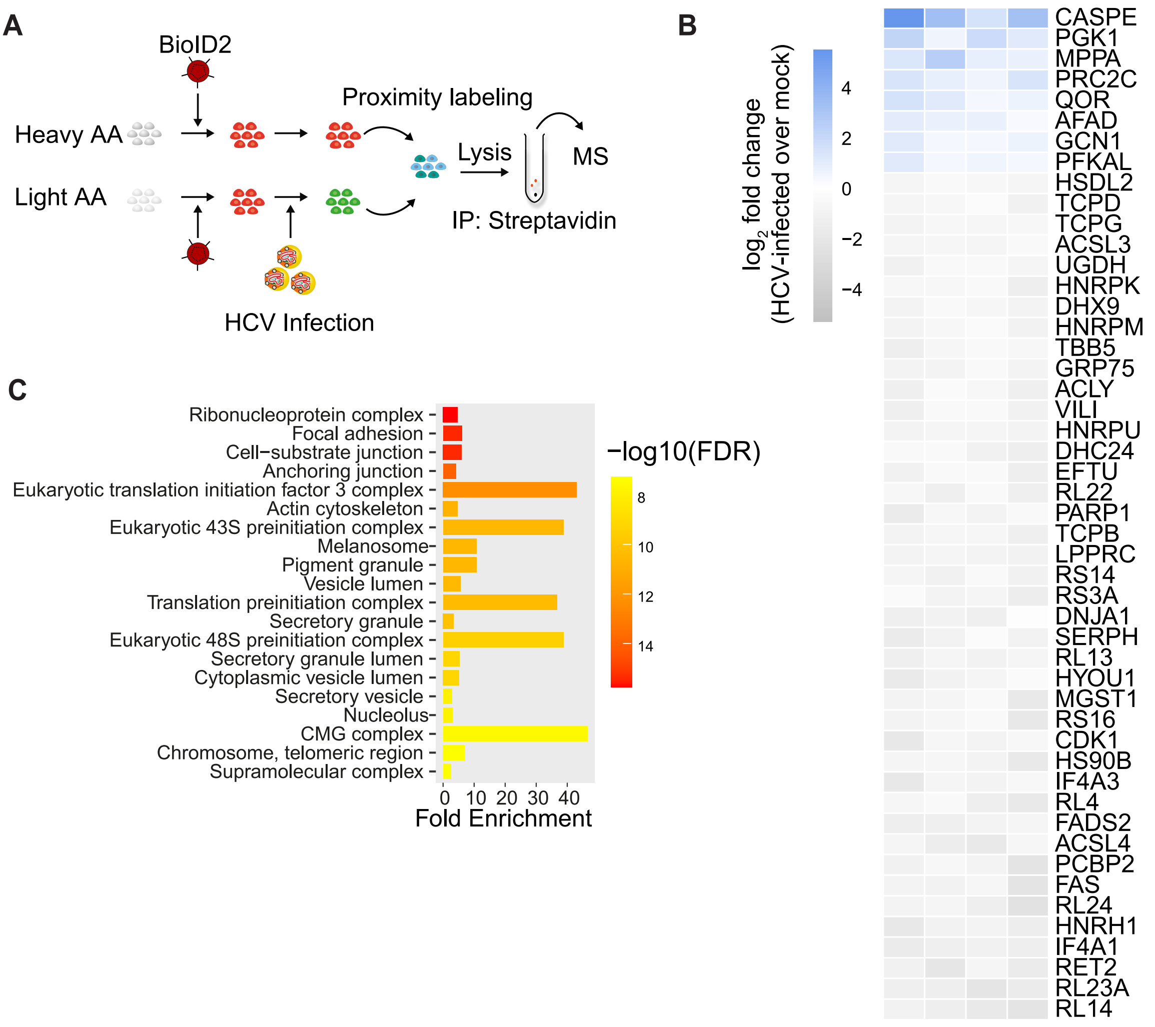

Figure S1: Identification of BioID2-proximal proteins in HCV-infected cells. (A) Scheme of the experiment. Huh7.5 cells cultured in media containing heavy or light amino acids were transduced with lentivirus expressing BioID2 and infected with a Jc1$^{NS5AB\text{-}EGFP}$ reporter strain (MOI 0.01). 3 weeks post infection, cells were incubated with biotin, and biotinylated proteins were pulled down using streptavidin-conjugated agarose beads and subjected to MS. (B) Heatmap of proteins significantly enriched and depleted proximal to BioID2 in HCV-infected cells compared to control; columns represent independent experiments ($\log_2$ fold change > 0.5 or < -0.5, unpaired two tailed t-test with unequal variance (Welch's t-test) $p \leq 0.05$, n = 4). (C) GO analysis of BioID2-proximate proteins enriched or depleted in HCV-infected cells. Bar graph of top 30 overrepresented cellular components terms of enriched or depleted proteins ($\log_2$ fold change > 0.5 or < -0.5) with a *p*-value (FDR) cutoff of 0.05. Shown are the number of genes sorted by $\log_{10}$ (FDR).

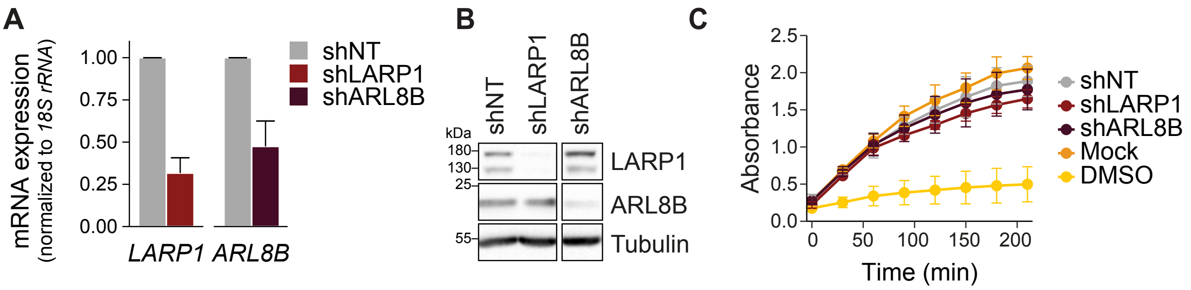

Figure S2: Validation of LARP1 and ARL8B shRNA. Huh7.5 cells were transduced with lentivirus coding for either shARL8B, shLARP1, or shNT (non-targeting). (A) Knockdown efficacy was verified by qRT-PCR. Shown is the relative expression of *ARL8B* and *LARP1* normalized to *18S rRNA*. Experiments were performed in duplicates (Mean ± SD, n_{ARL8B} = 3, n_{LARP1} = 2). (B) Protein levels of ARL8B and LARP1 were determined by immunoblot analysis. Tubulin served as loading control. Shown is one representative experiment (n = 3). (C) Viability assay of shRNA-transduced and control cells (Mean ± SD, n = 4).

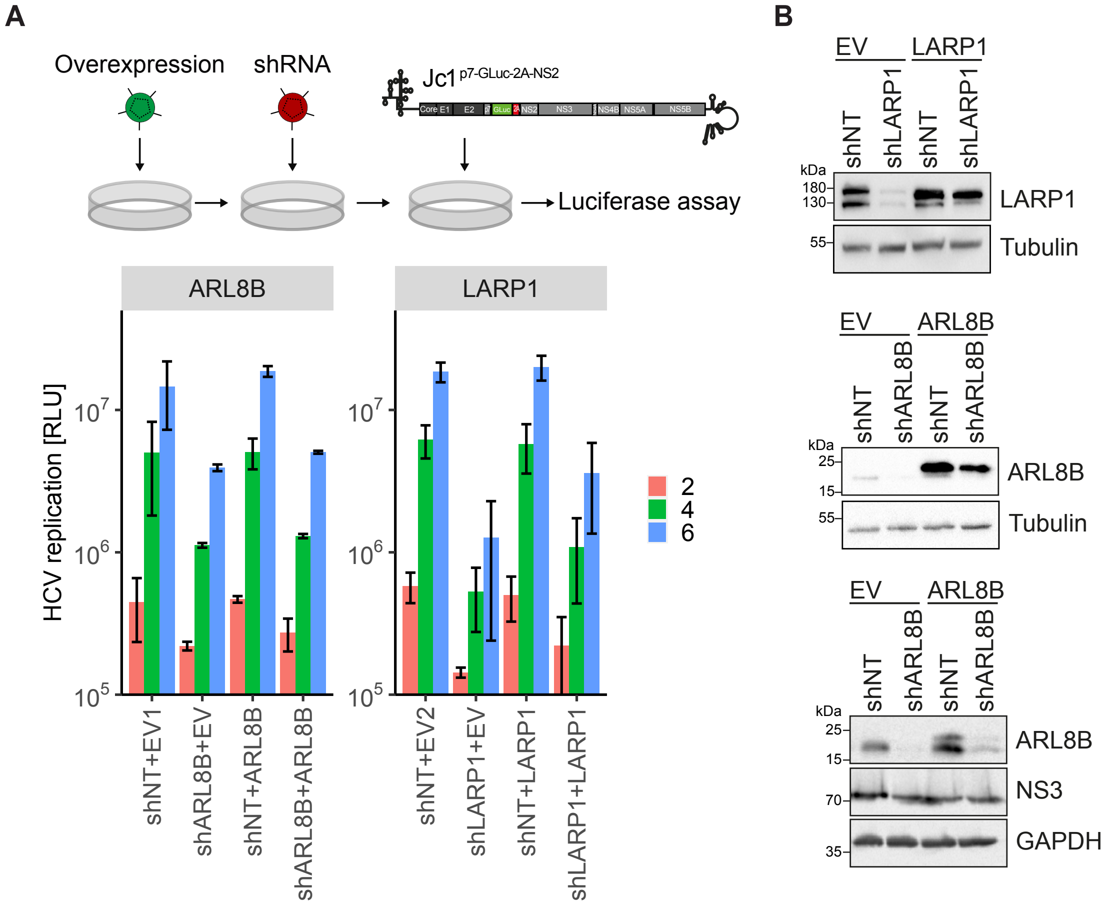

Figure S3: LARP1 overexpression partially rescues its effect on HCV replication in shLARP1 cells. (A) Huh7.5 cells were transduced with lentivirus expressing LARP1, ARL8B, or an empty vector and one day later transfected with shLARP1, shARL8B, or shNT, respectively. 3 days later cells were infected with Jc1$^{p7\text{-}GLuc\text{-}2A\text{-}NS2}$ (MOI 0.5) and luciferase activity in the supernatant was measured 2, 4, and 6 dpi. Shown are relative light units (RLU) (Mean ± SD, n = 2). (B) Protein levels of ARL8B and LARP1 in uninfected cells and protein levels of ARL8B in infected cells were determined by immunoblot analysis using the indicated antibodies. NS3 served as a control for infection. Tubulin or GAPDH served as loading controls. Shown is one representative experiment (LARP1 and ARL8B uninfected: n = 2; ARL8B infected: n = 3).

Table S1: Proteins identified through proximity labeling of ANXA3-BioID2.
Sheet “ANXA3-proximal proteins” shows the UniProt ID and Gene Name of the proteins identified in all four experiments (A–D) with the $\log_2$ enrichment ANXA3-BioID2 over BioID2 control, as well as the mean and the *p*-value.
Sheet “ANXA3-proximal proteins in HCV” shows the UniProt ID and Gene Name of the proteins identified in all four experiments (A–D) with the $\log_2$ enrichment HCV-infected over uninfected control, as well as the mean and the *p*-value.
Sheet “BioID2-proximal proteins in HCV” shows the UniProt ID and Gene Name of the proteins identified in all four experiments (A–D) with the $\log_2$ enrichment HCV-infected over uninfected control, as well as the mean and the *p*-value.
